# A combinatorial set of 3,125 cartoon characters based on five attributes for research on categorization, judgment, decision-making, and memory with adults and children

**DOI:** 10.3758/s13428-025-02758-4

**Published:** 2025-08-01

**Authors:** Arndt Bröder, Monika Undorf, Mihail Gututui

**Affiliations:** 1 School of Social Sciences, University of Mannheim, L13, 17, D- 68131 , Mannheim, Germany; 2 Technical University Darmstadt, Darmstadt, Germany; 3 University of Applied Sciences Mannheim, Mannheim, Germany

**Keywords:** Cognitive Psychology, Experimental stimuli, Categorization, Judgment, Decision Making

## Abstract

A combinatorial set of pictorial stimuli generated from five five-valued attributes is presented (and shared freely under the CC BY-NC-ND license, https://osf.io/dq2k8) which can be used in various cognitive research areas like categorization, multiple cue probability learning, judgment, decision-making, memory, or metamemory. The stimuli consist of five different cartoon characters combined with four five-valued attributes, namely five different hats, shoes, equipment, and jackets. The characters were created in a way to make them suitable for research with children and adults alike. In four studies, we assessed the similarity structure and attribute salience via similarity judgments (Study 1) and validated the extracted attribute salience in a judgment task (Study 2) and the similarity in a recognition memory task with adults (Study 3) and 6- to 8-year-old children (Study 4). Obtaining a random sample of 12,251 similarity ratings from 51 online participants in Study 1 allowed us to quantify the salience of attributes by analyzing perceived similarity via multilevel regression. We provide similarity values extrapolated from the regression model for all 4,881,152 stimulus pairs to allow for similarity-controlled stimulus selection. Study 2 validated the salience estimates for all attributes by showing their influence on learning speed and accuracy in a cue learning paradigm. Study 3 demonstrated the validity of the extrapolated similarity values by showing their impact on recognition performance, and Study 4 showed the suitability of the stimuli for research on children.

In experiments on categorization, function learning, multiple cue probability learning (MCPL), judgment and decision-making, or memory and metamemory, researchers often rely on stimuli that have a certain structure of *cues* or *attributes.* The relation between attributes and some outcome variable (e.g. category membership, numerical estimate, probability etc.) can often be defined in an arbitrary way to fulfill needs of testing cognitive models or judgment strategies. However, the stimuli are usually either verbal in nature or verbally described, have a very abstract nature, or have very limited attributes (for example, only binary attributes). The materials introduced here (a) provide more concrete nonverbal stimuli (that are created in a way to make them suitable for research with children), (b) contain five-valued cues making the selection more flexible, and (c) provide measures of the attributes’ impacts on perceived similarity (i.e., salience of attributes). These features allow researchers to flexibly select subsets of stimuli that are suitable for their respective research question. Figure 1 shows all five characters with all variants of hats, shoes, clothing, and equipment. Note that each of the attributes has five values, so there are 5^5^ = 3,125 stimuli altogether, comprising all combinations. In the extended stimulus set, each of the attributes can also be missing, which can be useful for memory experiments, for example.

**Fig. 1:**
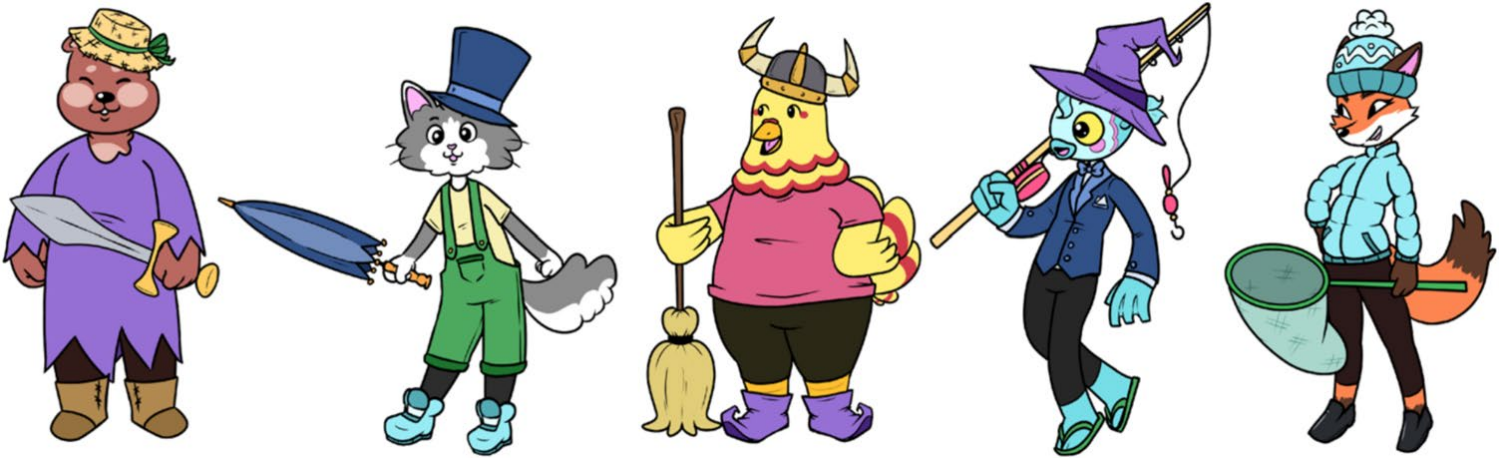
Five characters (bear, cat, chicken, fish, fox) with five types of hats (straw, top, viking, witch, bobble), shoes (leather boots, winter boots, witch shoes, flip flops, formal), equipment (sword, umbrella, broom, fishing rod, net) and clothing (witch robe, overall, t-shirt, tuxedo, winter jacket). Characters and attributes © Mihail Gututui

In this article, we will first outline the motivation for developing the stimuli based on the cognitive literature. Next, we will describe our stimuli, and the naming conventions used in the stimulus set. Third, we will describe three empirical studies: The first study used pairwise similarity judgments to establish a similarity structure as well as the salience of all attributes. Since (perceptual) similarity is an important determinant of categorization and judgment processes and respective models, the possibility to select stimuli according to similarity criteria may be useful for many researchers. The analysis also yields *salience* values for all attributes (and attribute values) which allow for manipulating this aspect in studies as well. Hence, one may create sets of attribute patterns that are formally equivalent with respect to a task or a model whereas the salience patterns differ. For instance, in a category learning experiment, category membership might be described by a formal rule, for example a conjunction rule stating that both Cue Value X and Cue Value Y jointly determine membership in an arbitrary category. The same rule may apply to either salient or less salient cues in different experimental conditions, so that researchers may examine the impact of salience independent from the complexity of the classification rule. We demonstrate the validity of the salience values in a judgment learning task in Study 2. Studies 3 and 4 demonstrate the validity of the extrapolated similarity norms in recognition tasks for adults and children. In the final section, we describe ideas for research questions that can be tackled with the stimuli.

## Motivation: attribute patterns as an input to many formal models

In many fields of Cognitive Psychology, theories and models have reached a high level of formalization (e.g. Farrell & Lewandowsky, 2018). On an abstract level, formal models of categorization, judgment, decision-making, multiple cue probability learning, and function learning assume that stimuli are defined by various attributes*.* Depending on the research tradition, these stimulus aspects may also be named *dimensions, features,* or *cues*. The cognitive processes in formalized models typically operate on these attributes and integrate them according to some logical or mathematical function to generate a behavioral response.

For example, in decision-making research, many strategies and heuristics have been proposed for how people integrate attribute information. The lexicographic rule, for example, compares decision options on the most important attribute and chooses the one with a superior value. In case of no (sufficient) difference between the attribute values, the next most important attribute is compared, and so on (Gigerenzer & Goldstein, 1996; Payne et al., 1993). Other strategies such as the equal weights rule or the weighted additive rule linearly combine all available attribute values (Payne et al., 1993). Another class of models formally describes information integration as a parallel constraint satisfaction process operating on a network representation of options and attributes (Glöckner & Betsch, 2008). The common denominator here is that all models describe algorithms specifying how attribute patterns map on responses.

To give another example, models of categorization or judgment predict behavioral responses from *similarities* between the to-be-judged stimulus and exemplars (or a prototype) in memory. These similarities are formalized as functions of the attributes defining the stimuli (e.g. Medin & Schaffer, 1978; Nosofsky, 1984). In Nosofsky’s Generalized Context Model, for example, the attributes are dimensions in a multidimensional psychological space and similarity between options is an exponentially decreasing function of Euclidean distance in this space.

As a final example, multiple cue probability learning (MCPL) studies present stimuli comprised of multiple cues, and people learn by repeated feedback to predict some criterion value (e.g., an event probability). There is a "correct" value of the criterion defined by some formal function of the cue patterns, and the speed of learning depends on the complexity of this rule (Little & Lewandowsky, 2012).

Hence, in different areas of Cognitive Psychology, either the cognitive models, the experimental paradigms, or both demand stimuli with a clearly defined attribute structure that can be related by the experimenter to a criterion value (e.g. category, quantitative attribute, prediction) in an arbitrary way.

## Motivation: Creating versatile and concrete nonverbal stimuli

It is of course not possible to enumerate all kinds of stimuli that have been used in the literature so far, but we want to give some flavor here: On the one end of the spectrum are studies of categorization that use very abstract stimuli like geometrical shapes with varying attributes (size, length, color) as input (e.g. Donkin et al., 2015; Goldstone, 1994; Medin & Schaffer, 1978; Nosofsky, 1986). This has the advantage of excluding all semantic influences and most influences of prior knowledge, and the formal relation between category membership and attributes can easily be defined and manipulated to derive different category structures (e.g., Shepard et al., 1961).

At the other end of the spectrum are real-world stimuli for which the stimulus features first have to be extracted, such as by running multidimensional scaling methods on large numbers of similarity judgments obtained from independent raters. For example, Nosofsky et al. (2018c) obtained feature space representations of a large collection of rocks and minerals and used them to test formal models of categorization learning in this natural science domain (Nosofsky et al., 2018a, 2018b, 2019). In a similar endeavor on a smaller scale, Izydorczyk and Bröder (2023) obtained feature space representations for a judgment domain involving 32 different bird species. Although this work is important for transferring cognitive modeling to real-world domains (and thereby also using more engaging stimuli), this approach comes with two downsides when viewed from a basic science perspective: First, any real-world domain may involve unknown amounts of participants’ prior knowledge that are not under the experimenter’s control. Second, natural stimuli may contain numerous judgment-relevant features in addition to those that were extracted by multidimensional scaling procedures. If so, people’s behavior may be influenced by exogenous factors not captured by the modeled attributes. Poor model fits may then result from missing out relevant cues rather than misspecified cognitive models.

Many judgment and decision-making studies have taken the middle ground between abstract geometrical shapes and real-world stimuli by constructing more or less quasi-realistic stimuli that retain some advantages of both extreme ends of the spectrum: On the one hand, the stimuli are *only* defined by the attributes. Consequently, exogenous factors should be reduced to a minimum. Also, stimuli can be generated by combining all possible attribute patterns from the component attributes, and it is possible to define arbitrary functional relations between patterns and optimal responses. On the other hand, the stimuli presumably have more face validity and are more engaging and motivating than abstract geometrical shapes. In judgment research, for example, Juslin et al.’s (2003) “death bug” stimuli have gained some popularity. The cover story is to learn about the toxicity of venomous bugs that are constructed of binary cues (e.g. leg length, nose color, spots vs. no spots on back, buttock color, see Juslin et al., 2008). In most cases, the bugs are shown as pictures (Bergert & Nosofsky, 2007; Juslin et al., 2003, 2008; Nosofsky & Bergert, 2007), but verbal attribute lists have been used as well (Pachur & Olsson, 2012; Trippas & Pachur, 2019). Lists of verbal cue values have been also used in numerous other cover stories such as judging the severity of a tropical disease (Bröder et al., 2010; Gluck & Bower, 1988; Persson & Rieskamp, 2009), choosing between hypothetical stocks (Bröder, 2000; Newell & Shanks, 2003; Nilsson et al., 2005), predicting city size (Glöckner & Bröder, 2014; Juslin & Persson, 2002), or judging job candidates (Rosner & von Helversen, 2019; von Helversen et al., 2014). Examples of pictorial stimuli constructed from combining attributes are fictitious flowers judged for their selling price (Izydorczyk & Bröder, 2022), suspects in a hypothetical murder case (Bröder & Schiffer, 2003), fictitious butterflies (Hoffmann et al., 2016), or cartoon characters (Hoffmann et al., 2014, 2016).^1^ We do not claim any completeness concerning this exemplary list.

However, it is evident that some applications require quite sophisticated knowledge in order to make sense of the cover stories (e.g. job candidates, stock predictions, murder suspects, and even death bugs) that may produce unwanted differences between adult participants and exclude their use for research on children. Also, many labels are verbal which typically precludes pre-school children and complicates the use of stimuli by researchers working with participants who have different language backgrounds. Finally, many studies were published before the advent of the open science movement so that not all more complex stimuli are freely available.

The set of stimuli introduced here aims to provide researchers with several additional benefits: First, we aimed at (a) nonverbal stimuli that are usable and engaging for both adults and children that are (b) devoid of most intrinsic semantic constraints due to prior knowledge and (c) provide many combinations to choose appropriate subsets from. Second, these stimuli are well-documented and freely shared for interested researchers to use. Third, since many formal models of categorization and judgment involve *similarities* of stimuli as an important cognitive variable, we provide pairwise similarities and cue saliences extracted therefrom as additional information that enable researchers to select stimuli according to their needs.

## The stimuli

As described above, the stimuli comprise 5 cartoon characters (bear, cat, chicken, fish, fox) combined with 4 five-valued attributes (clothing, hat, equipment, shoes) which results in 5^5^ = 3,125 combinations.

### Licensing and availability

 The stimuli are licensed under the Creative Commons license CC BY-NC-ND, meaning that they may be freely used and shared for noncommercial purposes such as research, but may not be altered (except for image size), and that the creators must be credited. The latter should be done by citing the current article. The 3,125 stimuli, along with all documentation, similarity values, and analysis scripts of the validation studies reported below are available at https://osf.io/dq2k8.

### Naming convention and technical aspects

The stimuli are available as PNG graphics in high resolution with 300 pixels per inch (1,889 x 1,889 pixels) or smaller versions (about 590 x 590 pixels) with transparent background. They may be further reduced in size with appropriate software. The files are coded with numbers to reflect the stimulus composition ranging from file “BUG_11111.png” to “BUG_55555.png”. Table 1 shows the coding key of the characteristics. For example, the cat in Fig. 1 can be found in file BUG_23451.png (2 = cat, 3 = top hat, 4 = winter boots, 5 = umbrella, and 1 = overall, see Table 1). The coding allows appropriate stimulus selection for displaying in computerized experiments. Of course, for use with children, it may be customary to print the stimuli in booklets or on stimulus cards.

**Table 1:** Coding of stimulus attributes in file names

	Code number position & coded attribute				
Code	1 character	2 hat	3 shoes	4 equipment	5 clothing
1	bear	bobble hat	flip flops	broom	overall
2	cat	straw hat	formal	fishing rod	t-shirt
3	chicken	top hat	leather	net	tuxedo
4	fish	viking hat	winter boots	sword	winter jacket
5	fox	witch hat	witch shoes	umbrella	witch robe

In the extended stimulus set, missing attributes have a value of 0.

## Study 1: Similarity ratings

The goal of this norming study was to assess the visual salience of the five cues and the corresponding cue values as well as the similarity structure of the cartoon characters. This was done by analyzing a large random sample of similarity ratings provided for pairs of stimuli. Using a multilevel regression model, we could (a) measure visual salience via the impact of cues on perceived similarity (regression weights) and (b) provide predicted similarity values for all possible pairs of stimuli. Note that obtaining ratings for all (3,125^2^/2)−3,125/2 = 4,881,250 possible pairs would have been unfeasible. Hence, a large random sample was generated to extrapolate to the stimulus pairs for which we did not obtain ratings.

### Method

#### Procedure 

**Fig. 2:**
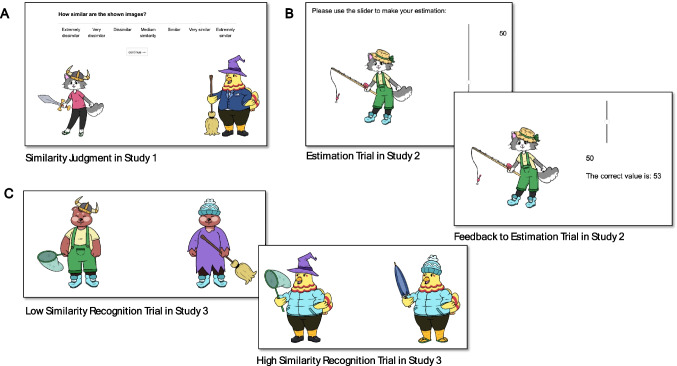
Screenshots of example trials in all studies. (**A**) Randomly paired stimuli were judged for similarity on a Likert scale, (**B**) Estimates were provided by adjusting the slider and changing the number, the correct number was provided on the feedback screen, (**C**) Two forced-choice recognition trials of Study 3

The study was programmed in *lab.js* (Henninger et al., 2022) and conducted online via Prolific.com. Instructions were in English, and we recruited residents of UK, USA, and Australia who were registered with English being their primary language. In addition, three student research assistants from our lab with good English skills who checked the programming were included since there was no reason to exclude their data. After a brief introduction, participants were presented two stimuli randomly drawn from the 3,125 cartoon characters together with a rating scale with the seven options “extremely dissimilar”, “very dissimilar”, “dissimilar”, “medium similarity”, “similar”, “very similar”, “extremely similar”, later coded with −3 to +3 for analysis. Each participant rated stimulus pairs for 10 minutes whereafter they could have an optional 1-minute break before rating stimulus pairs for further 10 minutes. They were told that the first 15 ratings were to familiarize them with the scale and the kind of stimuli. These judgments were discarded from the data. A screenshot of a random trial is shown in Fig. 2A.

#### Participants

We recruited three student assistants and 50 participants from the Prolific participant pool (UK, USA, Australia, primary language English). Two of the latter were excluded according to our predefined criterion that similarity ratings should at least correlate .30 with the number of attribute matches between stimuli. One of the excluded participants also claimed in a follow-up question that they did not respond seriously during the experiment. Hence, 51 participants constituted the final sample, with a mean age of 31.65 (range 18-41), 31 of which identified as female and 20 as male. Eight participants were students, 36 employed, seven marked "other".

### Results

The mean correlation between similarity ratings and the number of matching attributes was *r* = .59 (range .30 to .75), and the mean number of judgments provided by the participants was 255.41 (range 126 to 446). After erasing the first 15 judgments of each participant, we obtained 12,261 similarity judgments for 12,251 unique stimulus pairs (i.e. 10 pairs were judged twice).

**Fig. 3:**
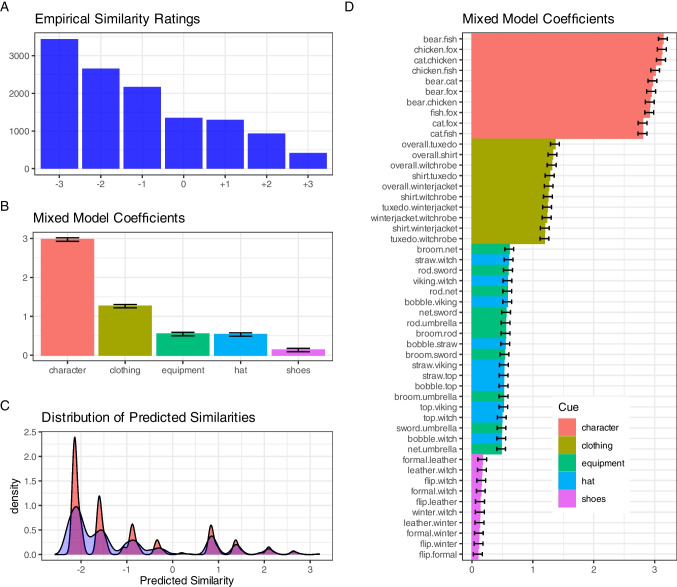
Similarity analysis of stimuli in Study 1. (**A**) Empirical frequency distribution of the similarity ratings showing a preponderance of relatively "dissimilar" judgments. (**B**) Mixed model coefficients for the regression analysis at the cue level with 95% CIs. (**C**) Density plot of predicted similarity values for all 4,881,250 stimulus pairs. The red curve shows the predictions from the cue model, the blue curve shows the predictions from the cue value model. (**D**) Mixed model coefficients for the regression analysis at the cue value level with 95% CIs. The coefficients denote how strongly a certain feature difference impacts on the dissimilarity of a pair

The mean rating was −1.09 (~ "dissimilar") with a standard deviation of 1.76 and a skew of 0.66. The empirical distribution of ratings is shown in Fig. 3A. The ratings were analyzed with multilevel regressions to investigate the impact of cues and cue matches on the perceived similarity of stimuli. One coarse-grained analysis analyzed this at the *cue level*, a more fine-grained analysis investigated the *cue value level.* We will explain both analyses in turn.

*Cue level.* In this analysis, we investigated how a *match* of values on a cue impacted similarity ratings. Five dummy-variables were created for the five cues character, clothing, equipment, hat, and shoes. These were coded as "0" when the values on a cue mismatched, but "1" if the values were equal (e.g., "hat.match" = 1 if the two cartoon characters wear the same kind of hat). The similarity ratings were analyzed with a multilevel linear regression with fixed effects for the predictors and random intercepts for participants using the *lme4* package (Bates et al., 2015) in the statistics environment R (R Core Team, 2024).^2^ This model was superior to a baseline model with only participant random effects (χ^2^(5) = 12,451) and a model assuming equal weights of the cues (χ^2^(4) = 7,434.2). Figure 3B shows the regression coefficients. The weights form a non-compensatory structure with the character as the most impactful attribute, followed by clothing. Equipment and hat are less predictive of similarity perception, and the impact of the shoes is significant, but negligible in size.

*Cue value level.* For the fine-grained analysis, we created 10 dummy variables for each cue (i.e. 50 altogether), representing the 10 possible contrasts between cue values. If a specific contrast was present in the two stimuli (e.g., hat = viking hat vs. bobble hat), the dummy variable was coded as "1" (e.g., "bobble.viking" = 1), or 0 otherwise. Hence, a match on this cue was the implicit reference category (all 10 cue variables coded 0). We ran an analogous regression model for the similarity ratings as before with these new 50 predictors. The regression weights can be seen in Fig. 3D, ordered by their size.^3^ Results show that although specific cue value constellations within a cue category vary somewhat in importance, the cue level clearly has a much stronger impact on similarity judgments.

### Discussion

The results generated by drawing a large random selection of stimulus pairs are promising and intuitive: As expected, the character itself was the most salient cue, followed by the clothing, then hat and equipment on equal footing, and shoes having the smallest influence on perceived similarity. Eyeballing the stimuli, this probably conforms to the area the attributes cover in the pictures. Notably, the variation between cues is much larger than the variation attributable to specific cue values within a cue (see Fig. 3D). Despite a relatively small sample, confidence intervals are very small, suggesting high consistency between the participants.

Using the models estimated from a large random sample of 12,251 unique cue constellations, we predicted the similarities of the remaining potential cue constellations and provide them in a csv file on the OSF repository. Figure 3C shows the density plot for the predicted values of the cue model (red) and the cue value model (blue) which correlate *r* = .995. On the OSF repository, we also provide a distance matrix, corresponding heat maps, as well as two multidimensional scaling results for 5 and 25 dimensions to allow for the selection of stimuli with different similarity structures.

To summarize, the quite consistent weighting of cues in similarity judgments across participants allowed us to establish a salience hierarchy of the five cues. Hence, stimuli can be selected so that cue salience is manipulated or controlled for. For example, Platzer and Bröder (2012, see also Bröder et al., 2021) manipulated the congruency between the cues's predictive validity and their salience, showing effects on decision strategy selection. Likewise, researchers in categorization, function learning, MCPL etc. might wish to vary the salience of predictive cues and can select stimuli accordingly from our set. Finally, since similarity is an important construct in computational models of cognition, one may use the predicted similarity values from our extrapolation to select sets of stimuli within certain similarity ranges.

## Study 2: Validation of cue salience with an MCPL task

The salience of cues assessed with the similarity ratings from Study 1 should impact the ease with which cue-criterion relationships are learned during an MCPL task. Salient cues should be detected more easily as predictors of a criterion than nonsalient cues. Hence, the goal of this study was to validate the salience values derived from Study 1 by demonstrating their impact on cue learning. To test this impact, we contrasted a congruent with an incongruent condition, following the logic of Bröder et al. (2021): In the congruent condition, the weights of cues determining the criterion value via a linear function had the same rank order as the saliences of cues assessed in Study 1. Hence, the most salient cue (character) was also the most important one, and the least salient cue (shoes) was the least important one. The rank order of cue weights was reversed in the incongruent condition, rendering the least salient cue (shoes) the most important one and the most salient cue (character) the least important one. The study was preregistered on https://osf.io/jux8e.

### Design and task

 A simple two-groups between-subjects design was used to contrast a congruent and an incongruent condition as described above. In addition, five counterbalancing subsets of 1,024 stimuli each were created by leaving out one value of each cue. This was done to make the task easier. Of each stimulus set (with four values for each attribute), two cue values were chosen randomly as positive instances (coded ‘1’) increasing the criterion value, the others were coded ‘0’ as negative instances. The cues predicted the criterion via Equation (1):

1 $$\begin{array}{l}\text{criterion }=\\ \text{round}((30*\text{Cue}1+20*\text{Cue}2+10*\text{Cue}3+5*\text{Cue}4+0*\text{Cue}5)*100/65)+\text{uniform}[-\text{3,3}] =\\ 46*\text{Cue}1+31*\text{Cue }2+15*\text{Cue}3+8*\text{Cue}4+0*\text{Cue}5+\text{uniform}[-\text{3,3}]\end{array}$$

In the congruent conditions, Cues 1 to 5 were “character”, “clothing”, ”hat”, ”equipment”, and “shoes”, respectively. In the incongruent condition, they were “shoes”, “hat”, “equipment”, “clothing”, and “character”.^4^

The participants’ task was to estimate the “social status” of creatures in a fictitious fairy-tale country named Lomiland. They gave an estimate and received feedback in each of 120 learning trials. 30 test trials without feedback followed. The experiment was programmed in *lab.js* (Henninger et al., 2022), and data were collected online via www.prolific.com.

### Participants

 Following the preregistered power calculations, we recruited 100 participants with residence in UK (82), the USA (11) or Australia (5) and English as their primary language. We expected to have 90 usable data sets after exclusions. Due to an unknown error, only 99 data sets were recorded by the JATOS server. Of these, 50 identified as male, 47 as female, and 2 as diverse/non-binary. 11 were students, 2 retired, 67 employed, and 19 checked “other”. The mean age was 36.13 (*SD* = 10.34, ranging from 18 to 63). They were paid 5.12 GBP for participation, and the 10 participants with best performance in the last 50 trials received an additional 0.5 GBP.

### Procedure

 Participants received a brief introduction to the Lomiland scenario and were shown five example cartoon characters with all attribute values appearing once (see Fig. 1). Depending on their counterbalancing condition, they then received additional information about the positive cue values of each cue in order to make learning easier. An example sentence is “A straw hat and a viking hat tend to go with higher prestige than a bobble hat or a witch hat”.

In each of the first 120 trials, one stimulus was randomly drawn from the respective counterbalancing set with the restriction that the criterion value was between 0 and 90. The stimulus was shown with a vertical slider next to it that could be adjusted to any value between 0 and 100. Participants provided their estimate with the slider, and they were provided with feedback reading “The correct value is: X” displayed next to their estimate with “X” replaced by the correct value. See Fig. 2 B, for an example screenshot. In the last 30 trials, no feedback was provided. These were composed of two sets of 10 stimuli with criterion values < 10 or > 90, respectively (for measuring extrapolation ability) and 10 stimuli with 10 < criterion < 90 as in the learning trials. Finally, participants provided demographic information and marked if they had worked on the task seriously and their data should be used. The mean time used for the task was 30.73 minutes (*SD* = 10.7).

### Hypotheses

 As preregistered, our main hypothesis was that learning of the linear rule would be hampered in the incongruent condition, showing as a lower performance in terms of accuracy in the learning trials (measured by the root mean squared deviations [RMSD] between judgment and criterion) and a slower improvement across four blocks of 30 trials each in the learning trials. Both would show as a main effect of condition and a block-by-condition interaction in a mixed ANOVA. A main effect of block should emerge as a sanity check that learning takes place in the MCPL task. As exploratory further analyses, we backed up the ANOVA with a multilevel regression on the absolute deviations between judgment and criterion in each trial, replacing the block factor with the centered trial number including all 150 trials.

### Results

#### Randomization

 The randomization used sampling with replacement and resulted in group sizes of 54 in the incongruent and 45 in the congruent conditions, respectively. The frequencies across the five counterbalancing conditions were reasonably uniform, χ^2^(4) = 2.47, *p* = .65, and they also did not vary across congruence conditions, χ^2^(4) = 3.56, *p* = .47.

#### Preregistered analysis

 In the preregistration, we defined as an exclusion criterion to eliminate all participants who showed a correlation of less than *r* = .20 between judgments and criterion in the last block of 30 study trials. However, this resulted in the exclusion of 35 participants, largely lop-sided with a higher dropout for the incongruent (28 of 54, 52%) than congruent (7 of 45, 16%) condition, χ^2^(1) = 12.61, *p* < .001. Hence, we report two analyses, one with and one without exclusion of participants.

**Fig. 4:**
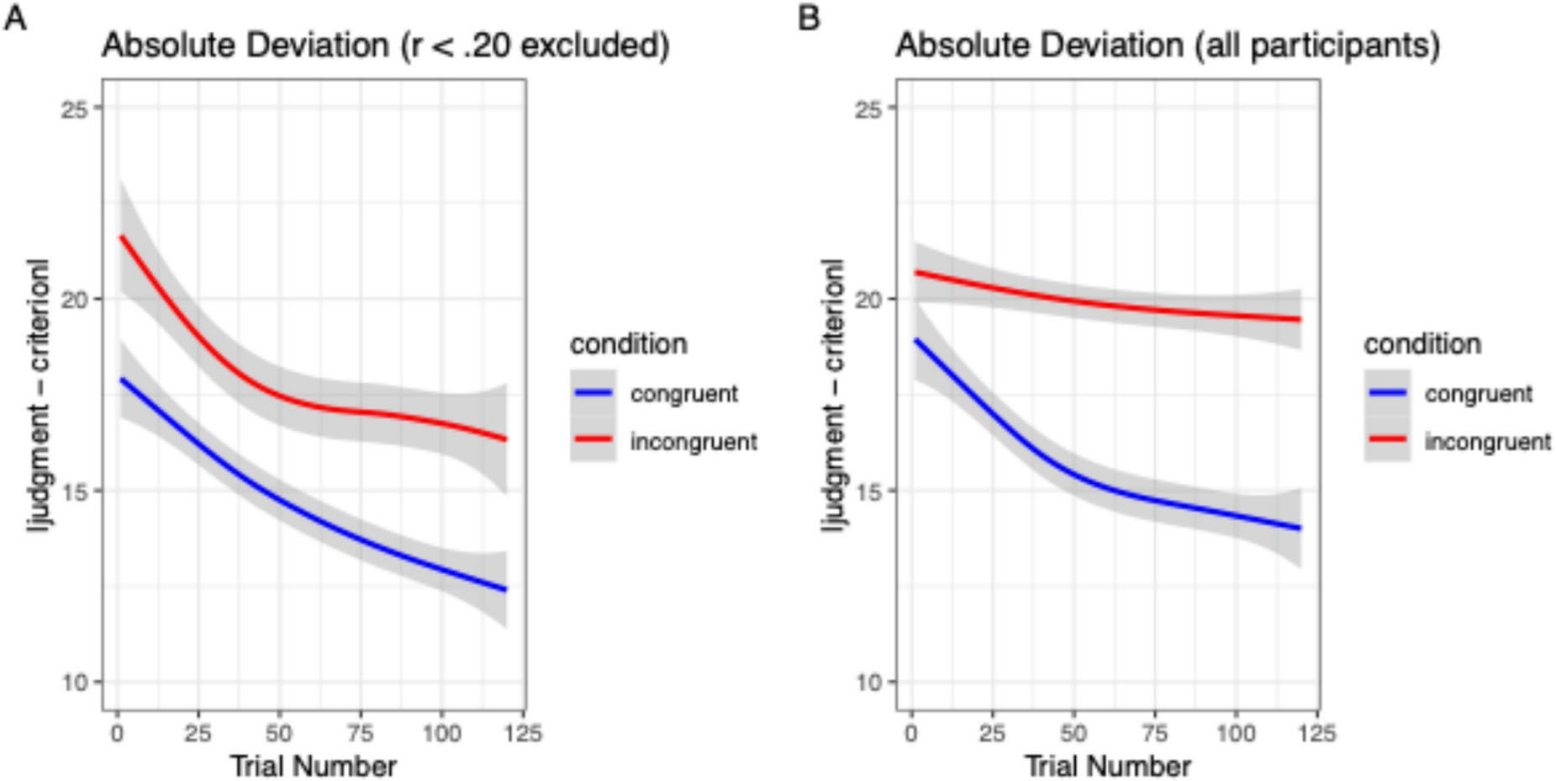
Trajectories of absolute deviations between judgments and correct value across the 120 learning trials. Panel (**A**) shows results with 35 participants excluded as preregistered; Panel (**B**) shows results with all participants

*Analysis with exclusion (as preregistered*). A mixed ANOVA of the RMSD (conducted using the *afex* package for R by Singmann et al., 2020) with the within-subjects factor *block* (1-4) and the between-subjects factor *condition* (congruent vs. incongruent) yielded significant main effects for both factors, *F*(1,168.93) = 36.06, *p* < .001, η^2^_p_ = .37, and *F*(1,62) = 13.32, *p* < .001, η^2^_p_ = .18, respectively. The interaction was not significant, *F*(1,168.93) = 1.02, *p* = .38. Panel A of Figure 4 visualizes the smoothed learning trajectory across all trials using the absolute deviation as an error measure.

#### Analysis without exclusions 

Since the preregistered exclusion criterion seemed overly strict and resulted in skewed exclusions, we reran the analysis with all cases included, replicating the main effects of condition, *F*(1,97) = 30.55, *p* < .001, η^2^_p_ = .24, and block, *F*(1,241.53) = 18.02, *p* < .001, η^2^_p_ = .163. Here, the interaction term also reached significance, *F*(1,241.53) = 5.30, *p* = .003, η^2^_p_ = .052, indicating more and quicker improvement in the congruent than the incongruent condition (see Panel B of Fig. 4).

Both analyses were also run using the correlations (rather than RMSD) as the dependent variables, and they yielded identical patterns of significance. The same was true for multilevel regressions with the absolute deviation per trial as the dependent variable and trial number rather than block number as a predictor (as well as random intercepts for stimuli and participants). We refer interested readers to the analyses on the OSF for details.

#### Additional analyses

 In the test trials without feedback, the full sample revealed much higher RMSD in the incongruent condition (*M* = 35.13, *SD =* 8.42), than in the congruent condition (*M* = 26.21, *SD =* 9.48), *F*(1,97) = 24.56, *p* < .001, η^2^_p_ = .20. The same qualitative result was obtained in the reduced sample after exclusions (*M*s = 23.74 and 28.91, *SD*s = 7.45 and 6.33, respectively, *F*(1,62) = 8.35, *p* = .005, η^2^_p_ = .12).

**Fig. 5:**
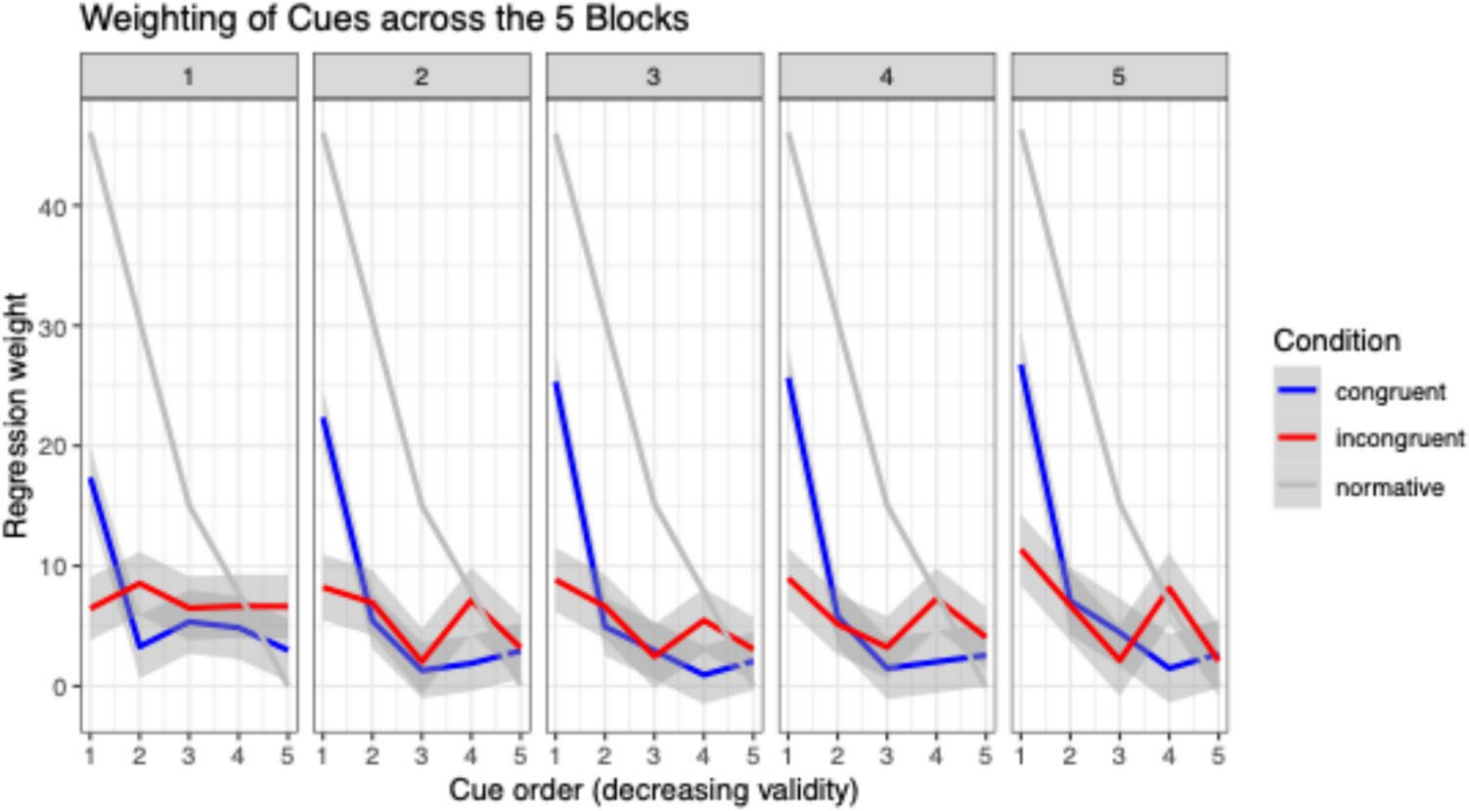
Regression weights for the five cues in descending order (x-axis) across the five blocks of trials in the experiment (panels). The light grey line ("normative") shows optimal weighting according to Equation (1)

Another fine-grained way to look at the data is to derive regression weights for the cues in each block for each participant by regressing judgments on the cues. Figure 5 shows five panels referring to each of the five blocks of trials in the experiment. We plot the mean regression weights for participants in both conditions along with the normative weights in each block. It can be seen that the congruent condition developed closer to the normative line although the weights are still far from optimal in Block 5. The change was much weaker in the incongruent condition. Also, it can be seen that the lion’s share of change was gained by figuring out the most important cue (Cue 1). Here, the difference between groups was most pronounced.

### Discussion

In Study 1, we interpreted the cue impacts on similarity ratings as measures of cue *salience*. To substantiate this claim, Study 2 investigated salience effects on cue learning by juxtaposing a congruent condition in which salience should be beneficial for figuring out the relative importance of cue weights with an incongruent condition in which salience should be rather detrimental. Although our preregistered exclusion criterion was too strict in hindsight and worked against our own block-by-condition interaction hypothesis, the main effect results in terms of RMSD, correlations, and individual regression weights all clearly showed that cue learning and accuracy were much better in the congruent than in the incongruent condition. Even the asymmetric exclusion itself showed that the incongruent condition was much harder (since participants with the lowest performance in the last block were excluded). Hence, despite the lack of a significant interaction (which pertains to the speed of learning) when asymmetric exclusion was done, we are very confident that our main research hypothesis was confirmed. A closer look at the regression weights reveals that in the current case, the main improvement was gained by figuring out the most important cue and giving it a high weight in the judgments (see Fig. 5).

## Study 3: Validating similarity values by recognition performance

The similarity between targets and distractors is a major determinant of memory performance in recognition tasks (e.g. Bayen et al., 1996; Kintsch, 1970; Klein & Arbuckle, 1970). Similarity ratings between stimuli were elicited only for a tiny fraction of possible stimulus pairs in Study 1, and similarity values for all possible pairs were extrapolated from the linear model in Fig. 2D. The third study was conducted to show that these extrapolated values have validity by predictably affecting memory performance in a 2AFC recognition test.

### Materials and design

 Each participant saw 15 stimuli in the study phase (recognition targets) that were paired with similar or dissimilar distractors in the final 2AFC recognition test. To counteract item effects of a specific set of stimuli, 10 sets were generated in the following way: For each set, 15 recognition targets were randomly sampled from the 3,125 stimuli with the restriction that each character (bear, cat, chicken, fish, fox) appeared three times in each set. For each of these targets, the most similar and the most dissimilar exemplar was chosen with the additional restriction that the character and one other attribute were identical while three attributes were different. Finally, no stimulus was allowed to appear twice in a set. For 7 randomly selected targets in each set, the most similar item was chosen as the distractor in the 2AFC recognition test, while the remaining 8 targets had the most dissimilar item as a distractor. Hence, our design entailed a simple within-participants comparison between similar vs. dissimilar target-distractor-pairs and a counterbalancing factor using ten different stimulus samples. The study was programmed in lab.js (Henninger et al., 2022).

### Participants

 As detailed in the preregistration of this study (https://osf.io/yxmjg), we targeted at 100 participants to reach 97 after exclusions for achieving a statistical power of 0.9 for detecting small-to medium effects (*d*_z_ = 0.3) in a one-tailed *t*-test with α =.05. We recruited 100 participants from UK, USA and Australia via Prolific.com who were at least 18 years old and had English as their primary language. One participant asked not to use their data for technical problems. In the remaining sample of 99, the mean age was 32.5 years (*SD* = 7.11, range 19-45 years), 45 participants were female, 53 identified as male, and one person identified as non-binary. Six participants reported occasional display problems (e.g., some stimuli loading slowly). We kept them in the sample and cross-checked results with omitting them. Doing so made no difference for any conclusions.

### Procedure

After giving consent to the study, participants were instructed to memorize 15 fictitious creatures and their outfits who would be shown sequentially on the screen. In the study phase, all targets of the randomly chosen set were shown in random order for 4 s each with a 500 ms ISI. After that, the participants answered three items from an unrelated risk perception scenario to erase recency effects. In the recognition phase, two creatures were presented next to each other (old and new stimulus randomly placed left or right in each trial), and participants were asked to denote which one was old by pressing "1" for the left or "0" for the right stimulus. Questions about age and gender as well as data quality followed. The experiment lasted between 4 and about 20 minutes. Participants were compensated with 1.20 GBP.

### Results

As preregistered, we conducted a simple one-tailed paired *t*-test to compare the percentages of correct choices for the similar and dissimilar pairs, respectively. As expected, the rate of correct choices was higher in the dissimilar condition (*M* = .73, *SD* = .19) than in the similar condition (*M* = .68, *SD* = .20), *t*(98) = 2.09, *p* = .019, (two-tailed *p* = .039), *d* = 0.21. The results do not change when participants reporting occasional display problems were excluded, *t*(92) = 2.42, *p* = .009, (two-tailed *p* = .017), *d* = 0.25, and a logistic mixed model regression with random intercepts for participants yielded the same result for both samples.

### Discussion

The second proof-of-concept study shows that the similarity values extrapolated from the judgments in Study 1 have psychological validity by affecting recognition memory performance in a predictable manner. Perceptually similar distractors hamper recognition of the targets. Given that the similarity values were all extrapolated from a model and not directly measured, this effect provides validating evidence in favor of the similarity values. The finding that the effect size was quite small can be explained by the relatively subtle similarity difference between the two conditions caused by our restrictive stimulus selection procedure where we forced equivalence on 2 attributes which led to mean similarity values of 0.87 and 2.01 in the dissimilar and similar conditions, respectively. Remember that the similarity rating scale used 7 bins from −3 to +3. Hence, our stimuli were rather on the similar side of the scale, and the difference between conditions was only 1.14 bins of the 7-point scale.

## Study 4: Pilot demonstration of suitability for child research

The stimuli were designed to be appropriate for research with children and adults alike. However, the first three validation studies investigated only adult samples. Hence, the goal of the fourth study was to demonstrate that the stimuli can be utilized for cognitive research on children as well. In particular, the mediocre recognition rates in Study 3 with only 15 stimuli raised the suspicion that the stimuli might be too difficult for children to remember.^5^

Using a convenience sample of *N =* 21 first and second graders who visited a German university for a Children’s University event, Lindow et al. (2025) aimed at conceptually replicating the results of Study 3 (similarity effect on recognition performance). Note that because of the small and much underpowered sample, we did not strive for statistical significance. Rather, we primarily wanted to demonstrate that the stimuli are well-accepted by children and suitable for research on children in principle.

### Materials and design 

All materials are available at https://osf.io/c5gb3/. A story was created in which the four characters Bela Bear, Frieda Fox, Kim Cat, and Hilda Hen (see OSF for German names) prepared their little house for a party. In three different scenarios (cleaning the house, catching fireflies for illumination and having the party), all characters were presented in front of clearly distinguishable visual backgrounds and with varying attributes. In each scenario, one attribute was constant across characters to signify the situation (broom for cleaning, net for catching, witch hat for partying). For the 2AFC recognition test, the children received a booklet with 12 pages. Each page displayed a formerly presented target character together with a distractor version of the same character. Distractors were either similar or dissimilar to the target (the independent variable varied within participants). In similar distractors, only one attribute was changed; in dissimilar distractors, two or three attributes were changed. There were two versions of the recognition test for counterbalancing the similarity condition of each target across participants.

### Participants

 *N* = 21 first- and second-year pupils (10 and 11, respectively) from an elementary school in Germany participated during an excursion to a university for science education. The mean age was 91.9 months (7.66 years, range 72 to 104 months), 12 children identified as female, 9 as male. Written consent to participate in the study was acquired beforehand from the participants’ parents or legal guardians. Children also stated informed consent.

### Procedure 

During a Children’s University event, participants were told to watch and listen to a story about four friends in Lomiland preparing a party. They were instructed to closely look at the characters in each situation who changed clothes or equipment between scenarios. The story was presented by projecting a presentation via a large format display. Each character was presented in each of the three scenarios, and a narrator read the story along with the presentation. After a very short retention interval of only a few sentences with general information on science, children were handed out the test booklets in which the target stimuli (characters as presented before) were shown with the respective scene background and along with a distractor in which one or more attributes were changed. Children were encouraged to mark the character they had seen before and to guess if they could not remember. Afterwards, booklets were collected, and data were coded. Booklets were then handed back to the children as a souvenir.

## Results and discussion

Unexpectedly, compared with the adult recognition results in Study 3, the recognition performance of the children was exceptionally good with an overall mean of 93% correct choices. There were even 9 children who did not make any errors across the 12 items. Despite this high overall performance level, the performance for items with similar distractors was significantly lower (90%) than for those with dissimilar distractors (96%), *t*(20) = −2.02, *p* = .03 (one-tailed), Cohen’s *d*_z_ = 0.44, confirming the expected difference.

This small study demonstrates that children between six and eight years could easily handle the stimuli, and they showed the well-documented effect of distractor similarity on recognition performance. The childrens’ memory performance was even considerably better than that of the adults in Study 3. Note, however, that Study 3 involved 15 memory targets and lacked the retrieval cues provided by the story and the different background layouts of the story’s three scenarios. According to informal observations by the experimenter, the story was highly engaging and motivating for the children, who followed the story presentation closely.

## Summary and general discussion

To summarize, our cartoon stimuli comprise a combinatorial non-verbal set of characters and attributes with pairwise similarity norms and a defined salience hierarchy of cues and cue values. The latter two aspects have been validated in Studies 2 to 4. Hence, researchers can choose stimulus configurations both on purely formal grounds (e.g., sets fulfilling certain statistical criteria with respect to attribute frequencies or correlations) or according to desired perceptual similarities (e.g., select very similar stimuli for one condition and very dissimilar stimuli for another condition).

The stimuli were explicitly designed to be used for samples with varying ages, cultural backgrounds, and levels of education. Importantly, meaning can be easily added by suitable cover stories, especially in research on children. For example, arbitrary semantic enrichment might be achieved by making attributes special: “Creatures carrying an umbrella have magic powers” or “Bears are allowed to live in the king’s palace”. This would be a simple way to manipulate attention to experimenter-chosen attributes, and it could be interesting to examine whether enriched features would have a memory advantage or attract increased attention in a categorization task. To mention a caveat, however, we do not claim neutrality of the stimuli across different cultures. The characters depicted might differ in familiarity or might evoke different associations and connotations across cultures. For example, bears or foxes may be less familiar in African or Asian cultures, and foxes might be associated with cleverness in one culture and wickedness in another. Hence, we recommend tailoring the stimulus use and particularly the semantic enrichment to the respective cultural background. Whether the stimuli work equally well across different cultures thus remains to be investigated.

Another advantage of the stimuli stems from their nonverbal nature: Collecting data from participants with diverse language backgrounds requires only minimal procedural adaptations to one’s experiments.^6^ The MCPL task in Study 2 and the recognition task in Studies 3 and 4 already illustrate the diversity of research questions for which the stimuli might be used. Also, different types of memory tasks are possible. Whereas we used a recognition task in which the whole attribute pattern had to be recognized, one may also use the extended set of stimuli for presenting characters with one (or several) missing attribute(s) and ask for their attributes in the study phase (e.g., “The bear forgot his hat at the birthday party. Do you remember what kind of hat he was wearing when he arrived?”). Similarly, one may think of studies in metamemory in which children predict their future memory performance, depending on the semantic enrichment of features (e.g., will they predict to remember the bear carrying a broom particularly well when being told that brooms grant magic powers?).

Another obvious research arena for using the stimuli is categorization research addressing exemplar-based or rule-based processes: Do children rather rely on similarity or on rules if they classify new stimuli after an extensive learning phase with training stimuli (e.g., Nosofsky, 1984)? Here, the similarity norms are helpful for generating predictions of exemplar-based processing and thus diagnose the kinds of processes people use. Similar questions about how cue integration can be tackled in the domain of multi-attribute decision-making where developing stimuli that are suitable for children has proven challenging (Betsch & Lang, 2013; Lindow & Betsch, 2018; Lindow et al., 2017).

We are aware that the demonstration of the stimuli’s suitability for preschoolers is still pending, but our fourth study with first and second graders as well as discussions with researchers in child development makes us optimistic that the stimuli are sufficiently engaging and can be embedded in stories that are suitable for preschoolers as well.

In conclusion, we introduce a novel stimulus set and hope that many researchers may find these stimuli useful, both as a versatile set for use in experiments in Cognitive Psychology and for developmental studies.

## Data Availability

Materials, data, and analysis scripts are freely available at https://osf.io/dq2k8/

## Footnotes

1 Hoffmann provides many of her stimuli at https://osf.io/wdfus/.

2 The model in *lme4* notation is: similarity.rating ~ (1|id)+character.match+hat.match+shoes.match+equipment.match+clothing.match

3 Note that mismatches predict similarity here, so all beta weights were negative. The absolute values are shown in Fig. 3D.

4 To illustrate this with the character depicted in Fig. 2B: The correct criterion value of "53" on the feedback screen might be composed by Cue1+Cue4-1 = 46+8-1 (the "-1" being a random term drawn from [-3,+3]). Hence, in both the congruent and incongruent condition, *cat* and *fishing rod* would be positive features contributing to the criterion ("social status"). However, the highly salient attribute "cat" is weighted 46 in the congruent condition, but only 8 in the incongruent condition. The less salient attribute *fishing rod* is weighted 8 in the congruent and 46 in the incongruent condition.

5 We thank an anonymous reviewer for pointing out this possibility.

6 With this statement, we do not dismiss the possibility that different linguistic or cultural backgrounds may affect how the stimuli are *processed*.

## Code availability

Analysis code available at https://osf.io/dq2k8/
